# The miR‐669a‐5p/G3BP/HDAC6/AKAP12 Axis Regulates Primary Cilia Length

**DOI:** 10.1002/advs.202305068

**Published:** 2023-12-13

**Authors:** Weina Wang, Xuyao Dai, Yue Li, Mo Li, Zongqi Chi, Xiaoyu Hu, Zhenshan Wang

**Affiliations:** ^1^ School of Life Sciences Institute of Life Science and Green Development Hebei University Baoding 071002 China; ^2^ School of Public Health Hebei University Baoding 071000 China

**Keywords:** AKAP12, G3BPs, HDAC6, miR‐669a‐5p, primary cilia

## Abstract

Primary cilia are conserved organelles in most mammalian cells, acting as “antennae” to sense external signals. Maintaining a physiological cilium length is required for cilium function. MicroRNAs (miRNAs) are potent gene expression regulators, and aberrant miRNA expression is closely associated with ciliopathies. However, how miRNAs modulate cilium length remains elusive. Here, using the calcium‐shock method and small RNA sequencing, a miRNA is identified, namely, miR‐669a‐5p, that is highly expressed in the cilia‐enriched noncellular fraction. It is shown that miR‐669a‐5p promotes cilium elongation but not cilium formation in cultured cells. Mechanistically, it is demonstrated that miR‐669a‐5p represses ras‐GTPase‐activating protein SH3‐domain‐binding protein (G3BP) expression to inhibit histone deacetylase 6 (HDAC6) expression, which further upregulates A‐kinase anchor protein 12 (AKAP12) expression. This effect ultimately blocks cilia disassembly and leads to greater cilium length, which can be restored to wild‐type lengths by either upregulating HDAC6 or downregulating AKAP12. Collectively, these results elucidate a previously unidentified miR‐669a‐5p/G3BP/HDAC6/AKAP12 signaling pathway that regulates cilium length, providing potential pharmaceutical targets for treating ciliopathies.

## Graphical abstract

Primary cilia transduce extracellular stimuli into cellular responses. This study identifies a microRNA, namely, miR‐669a‐5p as a potent regulator of cilium elongation rather than cilium formation. Mechanically, miR‐669a‐5p targets G3BPs to inhibit HDAC6 expression, which further upregulates AKAP12 expression. Collectively, it is proposed that the miR‐669a‐5p/G3BP/HDAC6/AKAP12 axis is a promising pharmaceutical target for treating ciliopathies.

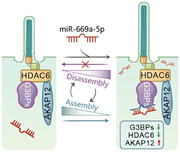

## 1 Introduction

Primary cilia (hereafter referred to as “cilia”) are membrane‐bound organelles that are found on most mammalian cells with microtubule protrusions and are derived from a parent centriole.^[^ ^1^ ^]^ Each cell contains a single cilium with evolutionarily conserved functions, acting as an “antenna” to sense and transmit extracellular signals into the cell. As such, cilia serve as initiators of a variety of signal transduction pathways and are necessary for cell development and physiological activities.^[^ ^2^ ^]^ Cilia are usually formed during the G1/G0 phase of the cell cycle and are absorbed before mitosis. Cilium length is a prerequisite for cilium function, and it is determined by two counteracting processes, namely, cilium assembly and disassembly. When the assembly rate is faster, the cilia are elongated; when the disassembly rate is faster, the cilia are shortened. Thus, the balance between assembly and disassembly is pivotal for maintaining regular cilium length.

Cilium length is extremely sensitive to external stimuli.^[^ ^3^ ^]^ For instance, leptin treatment increases the cilium length of hypothalamic neuronal cells;^[^ ^4^ ^]^ insulin mediates negative feedback mechanisms that regulate cilium length during adipogenic differentiation;^[^ ^5^ ^]^ and cilia on renal tubular epithelial cells are shortened upon water deprivation.^[^ ^6^ ^]^ A recent study elegantly demonstrated that cilium length in the hypothalamus undergoes periodic changes, which are associated with circadian rhythms.^[^ ^7^ ^]^ Dysregulation of cilium length causes multiple types of organ dysfunction, leading to a class of diseases called ciliopathies.^[^ ^8^ ^]^ Therefore, understanding how cilium length is regulated will provide new perspectives for the treatment and prevention of ciliopathies.

Cilia have been shown to function as sensors of environmental stress, and their length and functions are epigenetically regulated by microRNAs (miRNAs).^[^ ^9^ ^]^ miRNAs are small, noncoding RNAs approximately 22 nucleotides in length. They regulate gene expression at the posttranscriptional level by binding to specific RNA targets.^[^ ^10^ ^]^ miRNAs play essential roles in cell differentiation, proliferation, and apoptosis,^[^ ^11^ ^]^ and they profoundly affect the body's stress response to environmental changes. Hence, miRNA dysfunction is a major contributor to disease development.^[^ ^12^ ^]^ Compared with other classes of gene expression regulators, miRNAs meticulously regulate gene expression,^[^ ^13^ ^]^ and this delicate regulation is thought to be critical for maintaining physiological cilium length. However, little is known about how miRNAs regulate cilium length. Reportedly, miRNAs are highly expressed and meticulously regulate gene expression in ciliated cells.^[^ ^14^ ^]^ The miRNAs that are involved in cilium biology include those that specifically regulate cilium formation^[^ ^15^ ^]^ and those that regulate both cilium formation and elongation.^[^ ^9^, ^16^ ^]^ However, miRNAs that specifically regulate cilium length have rarely been reported. Given the multifaceted roles of miRNAs, we reason that miRNAs are potent regulators of cilium length, and the identification of those that regulate cilium length will provide novel therapeutic strategies for treating ciliopathies.

The screening of miRNA binding sites in cilium proteins has been the basis of research that aims to identify regulatory miRNAs in cilia.^[^ ^16^ ^]^ However, application of this approach has been challenging because cilium proteins are difficult to isolate and characterize. Thus, miRNAs and related regulatory axes in cilia have remained largely unexplored. Recently, a high‐throughput method for isolating cilium proteins has been reported.^[^ ^17^ ^]^ We speculate that efficient isolation of cilium proteins combined with small RNA sequencing can significantly expedite the identification of miRNAs that are involved in regulating cilium length.

Here, we report that a key miRNA, namely, miR‐669a‐5p, targets the stress granule proteins ras‐GTPase‐activating protein SH3‐domain‐binding proteins (G3BPs), which cooperate with histone deacetylase 6 (HDAC6) to regulate A‐kinase anchor protein 12 (AKAP12) expression levels, thereby regulating cilium length. This study provides a new therapeutic target and describes the implications for treating ciliopathies that are caused by abnormal cilium length.

## 2 Results

### 2.1 Identification of a miRNA That Regulates Cilium Elongation

**Figure 1:**
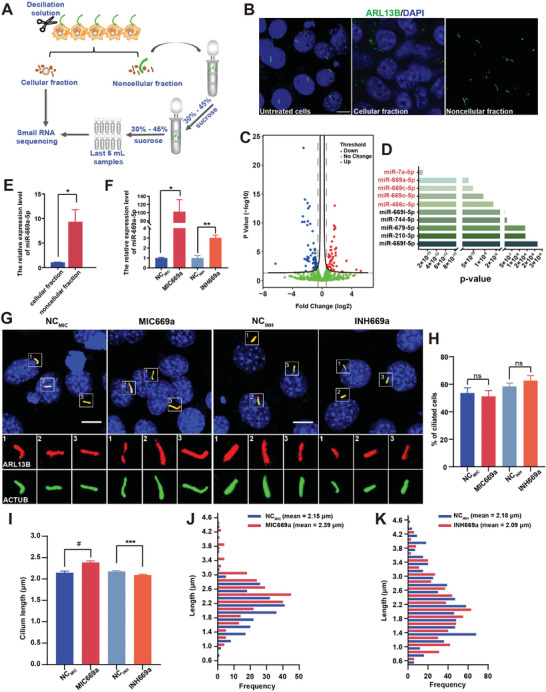
miR‐669a‐5p regulates cilium length rather than cilium formation in cells. A) Schematic diagram of cilium isolation by the calcium‐shock method. B) Representative images of the cells that were stained with anti‐ARL13B (green) antibody, and DAPI (blue). Scale bar, 5 µm. C) Volcano plot of differentially expressed miRNAs between cellular and noncellular fractions. The upregulated miRNAs (> two‐fold‐change, *p* < 0.05) and downregulated miRNAs (< 0.67‐fold‐change, *p* < 0.05) are highlighted in red and blue, respectively. Green spots indicate no significant change. D) The *p‐*value of differentially expressed miRNAs in the cellular and noncellular fractions of cells. E) Expression of miR‐669a‐5p revealed by qPCR analysis. *n* = 3 experiments. F) Expression of miR‐669a‐5p in cells transfected with miR‐669a‐5p NC (NC_MIC_) and mimics (MIC669a) or miR‐669a‐5p NC (NC_INH_) and inhibitors (INH669a), and starved for 24 h, as revealed by qPCR analysis. *n* = 4 experiments. G–K) Cells transfected with NC_MIC_, MIC669a, NC_INH_, or INH669a were starved for 24 h followed by staining with anti‐ARL13B (red) and anti‐AC‐α‐Tubulin (ACTUB, green) antibodies. DAPI (blue) was used to stain nuclei. Scale bar, 5 µm. H) Percentage of ciliated cells in (G). I**–**K) Quantitative analysis of cilium length in (G). *n* = 3, a minimum of 150 cells were used for each group. All data were presented as mean ± SEM; ns, not significant, ^*^ *p* < 0.05, ^**^ *p* < 0.01, ^***^ *p* < 0.001 and #*p* < 0.0001 (“#” represented “^****^”) by Student's *t*‐test or one‐way ANOVA and Bonferroni pairwise comparisons.

To investigate the molecular basis underlying the regulation of cilia structures and functions by miRNAs, we applied the calcium‐shock method, which was previously used to isolate cilia proteins from mammalian cells for proteomic analysis,^[^ ^17^ ^]^ to separate cilia fractions from NIH3T3 cell bodies (**Figure** **1**A). The cultured cells were fractionated into cellular and noncellular fractions, and immunofluorescence analysis (IFA) revealed that cilia were enriched in the noncellular fraction and were barely found in the cellular fraction (Figure 1B). Next, we performed small RNA sequencing with both fractions to identify cilium‐associated miRNAs. In total, 969 miRNAs were identified in both fractions (Table S1, Supporting Information). Of these, 42 miRNAs were significantly upregulated and 59 were downregulated in the noncellular fraction relative to the cellular fraction (Figure 1C). Furthermore, we performed a cluster analysis of these 42 noncellular miRNAs and chose the top five miRNAs, miR‐7a‐5p, miR‐669a‐5p, miR‐669c‐5p, miR‐669o‐5p, and miR‐466c‐5p, that were most significantly upregulated for further investigation (Figure 1D). Interestingly, these five miRNAs have been implicated in cell responses to environmental stress,^[^ ^18^ ^]^ which echoes the primary function of cilia as sensors of exogenous stimuli.^[^ ^19^ ^]^ Therefore, we reasoned that these five miRNAs could be important regulators of ciliogenesis.

**Figure 2:**
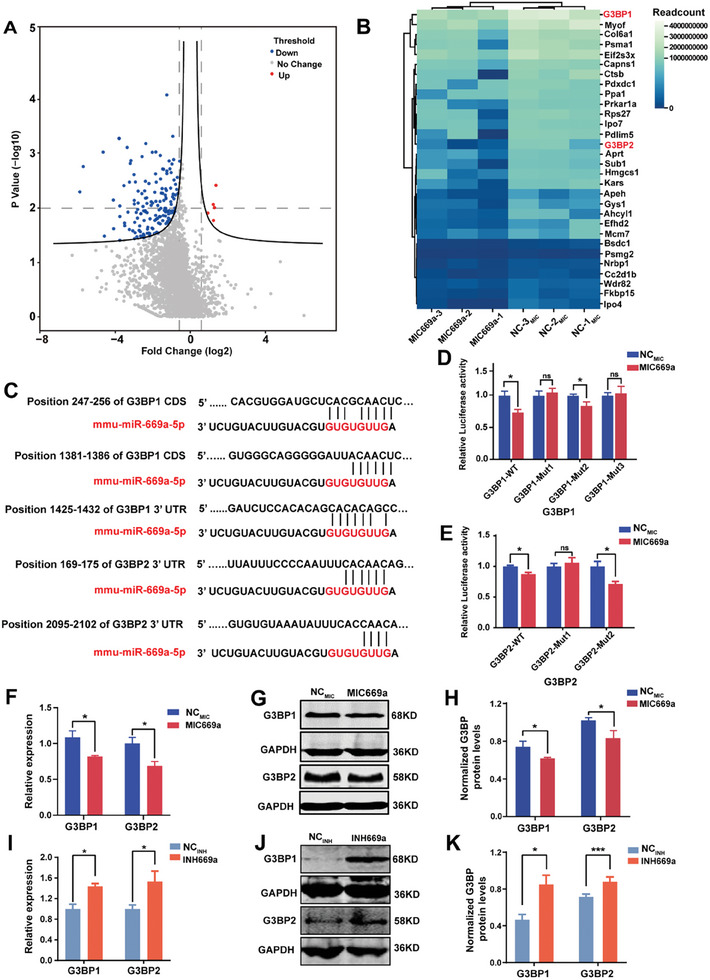
miRNA‐669a‐5p targets G3BPs and directly represses their expressions. A) Volcano plot of differentially expressed proteins between cells treated with NC_MIC_ or MIC669a. The upregulated and downregulated proteins (upregulated > 1.5‐fold‐change or downregulated < 0.67‐fold‐change, *p* < 0.05) were highlighted in red and blue, respectively. Gray spots indicate no differences in protein expression levels. B) Heatmap showing the top 30 enriched proteins among downregulated proteins in the noncellular fraction compared with the cellular fraction revealed by LC‐MS/MS (*n* = 2 experiments; fold change > 2). C) Schematics of the predicted miR‐669a‐5p binding sites of *G3BP1* and *G3BP2*. The seed sequences were shown in red. D,E) The luciferase activities were measured in cells that were transfected with *G3BP1* CDS or 3′UTR wild‐type (*G3BP1‐*WT) or mutated sequences (*G3BP1‐*Mut1, *G3BP1‐*Mut2, and *G3BP1‐*Mut3) together with NC_MIC_ or MIC669a (D). The luciferase activities measured in cells transfected with *G3BP2* 3′UTR wild‐type (*G3BP2‐*WT) or mutated sequences (*G3BP2‐*Mut1, *G3BP2‐*Mut2) together with NC_MIC_ or MIC669a (E). *n* = 5 from six independent experiments. F) qPCR analysis of *G3BP1* and *G3BP2* transcript levels after NC_MIC_ or MIC669a treatment. *n* = 4 groups from four independent experiments. G,H) Western blot probing with anti‐G3BP1 and anti‐G3BP2 in cells that were treated with NC_MIC_ or MIC669a. GAPDH was used as a loading control. *n* = 3 groups from three independent experiments; **I)**. qPCR analysis of *G3BP1* and *G3BP2* transcript levels after NC_INH_ or INH669a treatment. *n* = 4 groups from four independent experiments. J,K) Western blot probing with anti‐G3BP1 and anti‐G3BP2 in the cells that were treated with NC_INH_ or INH669a. GAPDH was used as a loading control. *n* = 3 groups from three independent experiments. All data were presented as mean ± SEM; ns, not significant, ^*^ *p* < 0.05 and ^***^ *p* < 0.001 by Student's *t*‐test or one‐way ANOVA and Bonferroni pairwise comparisons.

We then performed qPCR to verify the upregulation of miR‐7a‐5p, miR‐669a‐5p, miR‐669c‐5p, miR‐669o‐5p, and miR‐466c‐5p in the noncellular fraction. The results showed that the expression levels of miR‐7a‐5p, miR‐669a‐5p, miR‐669o‐5p, and miR‐466c‐5p were significantly higher in the noncellular fraction than in the cellular fraction (Figure 1E; Figure S1A, Supporting Information). However, miR‐669c‐5p expression was not significantly different between the two fractions (Figure S1A, Supporting Information). To further assess whether miR‐7a‐5p, miR‐669a‐5p, miR‐669o‐5p, and miR‐466c‐5p function in ciliogenesis, we transfected cells with validated random sequences as negative control (NC), mimics or inhibitors of the above four miRNAs. Compared to the NC group, the miR‐7a‐5p, miR‐669o‐5p, and miR‐466c‐5p inhibitors and mimics were effective in downregulating and upregulating their expressions, respectively (Figure S1B,C, Supporting Information). Moreover, miR‐669a‐5p mimics significantly increased the expression of miR‐669a‐5p, while the inhibitor appeared to be ineffective in silencing miR‐669a‐5p expression, as revealed by qPCR (Figure 1F). Reportedly, miRNA‐sponges can act as miRNA inhibitors,^[^ ^20^ ^]^ which prompted us to use miR‐669a‐5p‐sponges to inhibit miR‐669a‐5p expression. However, we observed that, instead of downregulating its expression, the application of miR‐669a‐5p‐sponges significantly increased miR‐669a‐5p levels. This unexpected result hints that miR‐669a‐5p‐sponges may function as a reservoir for miR‐669a‐5p instead by binding and enriching miR‐669a‐5p.^[^ ^21^ ^]^ Nevertheless, the effectiveness of the miR‐669a‐5p inhibitors could be verified by IFA and western blot results (Figures 1G−I and 2I–K).

To determine the regulatory role of miR‐7a‐5p, miR‐669a‐5p, miR‐669o‐5p, and miR‐466c‐5p in ciliogenesis, we analyzed cilium formation and measured the cilium length of cells that were transfected with miRNA inhibitors or mimics. Compared with the NC group, neither miR‐7a‐5p, miR‐669o‐5p, and miR‐466c‐5p inhibitors nor mimics changed the percentage of ciliated cells and cilium length (Figure S1D–F, Supporting Information), indicating that miR‐7a‐5p, miR‐669o‐5p, and miR‐466c‐5p are not involved in ciliogenesis. Similarly, neither miR‐669a‐5p inhibitors nor mimics could change the percentage of ciliated cells, suggesting that miR‐669a‐5p is not involved in cilium formation (Figure 1G,H). In contrast, we found that miR‐669a‐5p mimics effectively promoted cilium elongation (Figure 1G,I), resulting in an average cilium length of 2.39 ± 0.03 µm (miR‐669a‐5p mimic‐treated cells) versus 2.15 ± 0.03 µm (NC‐treated cells). Moreover, miR‐669a‐5p mimics significantly increased the frequency of cilia longer than 2.39 µm (Figure 1J). In addition, we observed that miR‐669a‐5p inhibitors effectively suppressed cilium elongation (Figure 1G,I), leading to an average cilium length of 2.09 ± 0.03 µm (miR‐669a‐5p inhibitor‐treated cells) versus 2.18 ± 0.03 µm (NC‐treated cells) and significantly increasing the frequency of cilia shorter than 2.09 µm (Figure 1K). Altogether, these results demonstrate that miR‐669a‐5p is a regulator of cilium length.

In most cells, ciliogenesis is dynamically regulated during cell cycle progression. Cilia are usually assembled in G0/G1 phase and disassembled immediately before mitosis.^[^ ^22^ ^]^ In our case, it is possible that the regulation of cilia by miR‐669a‐5p is synchronized with the cell cycle. To test this possibility, we stained the cells transfected with miR‐669a‐5p NC or mimics with DAPI and performed a fluorescence‐activated cell sorter (FACS)‐based cell cycle analysis. The data suggest that miR‐669a‐5p regulates cilium elongation in a cell cycle‐independent manner (Figure S1G–I, Supporting Information).

### 2.2 miR‐669a‐5p Targets G3BPs and Directly Represses Their Expression

We investigated the mechanisms underlying the regulation of cilium length by miR‐669a‐5p. First, we conducted a proteomic analysis of cells that were transfected with miR‐669a‐5p NC or mimics to identify the genes that are targeted by miR‐669a‐5p. In total, we found 326 differentially expressed proteins between the two groups, of which five were significantly upregulated and 321 were significantly downregulated (**Figure** **2**A,B; Table S2, Supporting Information).

The cluster analysis of the 30 most substantially downregulated proteins and the prediction of TargetScan and miRwalk algorithms revealed that *G3BP1* and *G3BP2* were target genes of miR‐669a‐5p. The prediction identified three miR‐669a‐5p binding sites (nucleotides 247–256, 1381–1386 and 1425–1432) in the coding DNA sequence (CDS) or 3′UTR of *G3BP1*, and two miR‐669a‐5p binding sites (nucleotides 169–175 and 2095–2102) in the 3′UTR of *G3BP2* (Figure 2C). Collectively, these results indicate that both *G3BP1* and *G3BP2* are putative target genes of miR‐669a‐5p.

We then used a dual luciferase reporter assay to verify whether *G3BP1* and *G3BP2* are indeed target genes of miR‐669a‐5p. We cloned the CDS or 3′UTR of *G3BP1* and the 3′UTR of *G3BP2*, both of which contain the miR‐669a‐5p binding motif, into a luciferase vector (Figure S2A,B, Supporting Information). Subsequently, the vectors were transfected into cells with miR‐669a‐5p NC or mimics. Relative to the cells transfected with miR‐669a‐5p NC, the cells transfected with miR‐669a‐5p mimics exhibited significantly lower luciferase activity (Figure 2D,E; *G3BP1‐*WT + NC_MIC_ vs *G3BP1‐*WT + MIC669a; *G3BP2‐*WT + NC_MIC_ vs *G3BP2‐*WT + MIC669a).

To further verify the binding of miR‐669a‐5p to *G3BPs*, we individually mutated the three putative miR‐669a‐5p binding sites of *G3BP1* and the two sites of *G3BP2*. These vectors were transfected into cells together with miR‐669a‐5p NC or mimics. The luciferase activities of the cells that were transfected with *G3BP1‐*Mut1 + NC_MIC_ or *G3BP1‐*Mut1 + MIC669a, as well as those of the cells that were transfected with *G3BP1‐*Mut3 + NC_MIC_ or *G3BP1‐*Mut3 + MIC669a, were not significantly different (Figure 2D); these results indicated that mutation of the first and third binding sites of *G3BP1* eliminated the inhibitory effect of miR‐669a‐5p on *G3BP1*. Similarly, the luciferase activities between the cells that were transfected with *G3BP2*‐Mut1 + NC_MIC_ and those that were transfected with *G3BP2*‐Mut1 + MIC669a were similar; these results indicated that mutation of the first binding site in the 3′UTR of *G3BP2* abolished the inhibitory effect of miR‐669a‐5p on *G3BP2* (Figure 2E). These results indicate that miR‐669a‐5p inhibits G3BP expression by targeting the first and third binding sites of *G3BP1* and the first binding site in the 3′UTR of *G3BP2*. Finally, miR‐669a‐5p NC was cotransfected into cells together with *G3BP1* CDS, *G3BP1* 3′UTR, *G3BP2* 3′UTR, or the respective mutated sequences. No significant differences in luciferase activities were observed (Figure S2C,D, Supporting Information), indicating that the decreases in the luciferase activities did not occur due to mutations in the binding sites in *G3BPs*. In addition, both qPCR and western blot revealed that the expression levels of G3BP1 and G3BP2 were significantly lower in the cells that were transfected with miR‐669a‐5p mimics than in the cells that were transfected with miR‐669a‐5p NC (Figure 2F–H). Similarly, the expression levels of G3BP1 and G3BP2 in the cells transfected with miR‐669a‐5p inhibitors were significantly higher than those in the cells transfected with miR‐669a‐5p NC (Figure 2I–K). Collectively, our data demonstrate that miR‐669a‐5p directly targets *G3BPs* and inhibits their expression levels.

### 2.3 Loss of G3BPs Results in Abnormally Longer Cilia

**Figure 3:**
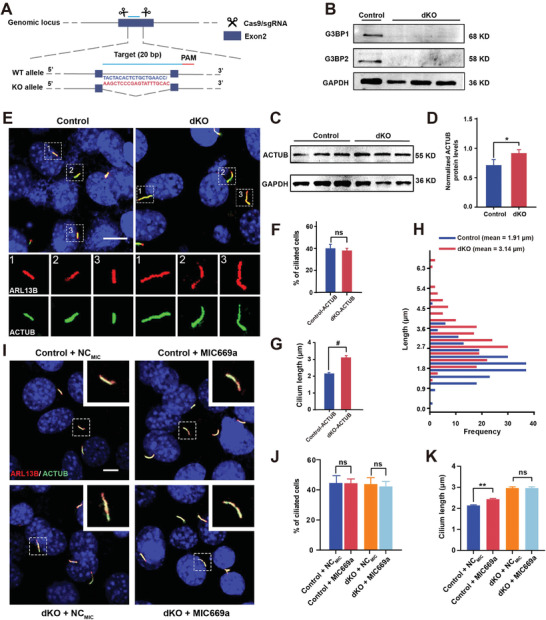
G3BP deletion promotes cilium elongation. A) GRISPR‐mediated targeting for *G3BP1* and *G3BP2* genes. G3BP1 and G3BP2 sgRNA sequences are shown in blue and red, respectively. B) Western blot probing with anti‐G3BP1 and anti‐G3BP2 in control and G3BP dKO cells. GAPDH was used as a loading control. *n* = 3 experiments. C,D) Western blot of cell lysates of control and G3BP dKO cells probed with anti‐ACTUB and GAPDH as a loading control. *n* = 3 from three independent experiments. E–H) Control and G3BP dKO cells were starved for 24 h and stained with anti‐ARL13B (red) and anti‐ACTUB (green) antibodies. DAPI (blue) was used to stain nuclei. Scale bar, 5 µm (E). Percentage of ciliated cells (F) and Quantitative analysis of cilium length (G,H) in control and G3BP dKO cells. I–K) Control or G3BP dKO cells treated with NC_MIC_ or MIC669a were starved for 24 h and stained with anti‐ARL13B (red) and anti‐ACTUB (green) antibodies. DAPI (blue) was used to stain nuclei. Scale bar, 5 µm (I). Percentage of ciliated cells (J) and quantitative analysis of cilium length (K) of MIC669a treated control and G3BP dKO cells. *n* = 3, a minimum of 150 cells were used for each group. All data were presented as mean ± SEM; ns, not significant, ^*^ *p* < 0.05, ^**^ *p* < 0.01 and #*p* < 0.0001 by Student's *t*‐test or one‐way ANOVA and Bonferroni pairwise comparisons.

G3BP1 has been reported to regulate ciliogenesis.^[^ ^23^ ^]^ Our results described above indicated that *G3BPs* were the target genes of miR‐669a‐5p and that overexpression of miR‐669a‐5p promoted cilium elongation. Therefore, we hypothesized that miR‐669a‐5p might regulate cilium length by targeting G3BPs. To test this hypothesis, we examined how cilium length changed upon loss of G3BP expression. We designed sgRNAs that target exon two2 of *G3BP1* and *G3BP2* and generated a double knockout (G3BP dKO) cell line using the CRISPR/Cas9 strategy (**Figure** **3**A,B).

Western blot analysis showed that the cilium marker, AC‐α‐Tubulin, was significantly upregulated in G3BP dKO cells compared with control cells (Figure 3C,D). Next, we quantified the percentage of ciliated cells and cilium length in the control and G3BP dKO groups by costaining for the cilium markers AC‐α‐Tubulin and ARL13B. The degree of cilia formation was indistinguishable between the control and G3BP dKO groups, whereas the cilium length of the G3BP dKO group was significantly longer than that of the control group (Figure 3E–H). The average cilium length of control cells was 1.91 ± 0.04 µm, while that of G3BP dKO cells was 3.14 ± 0.09 µm (Figure 3H). Similarly, compared with the control group, knockout of either G3BP1 or G3BP2 did not affect cilium formation, but cilium length was significantly increased (Figure S3A–J, Supporting Information). This result indicates that G3BP1 and G3BP2 do not affect cilium formation but regulate cilium length. In addition, the extent of cilium elongation in G3BP dKO cells was greater than that in G3BP1 KO or G3BP2 KO cells (Figure S3K,L, Supporting Information), suggesting that both G3BP1 and G3BP2 may regulate cilium elongation in parallel.

To confirm that miR‐669a‐5p regulates cilium length mainly via G3BPs, we quantified the percentage of ciliated cells and cilium length in G3BP dKO cells that were transfected with miR‐669a‐5p NC or mimics. We found that neither the formation nor the length of cilia was significantly different in both groups (Figure 3I–K). Taken together, our data confirm that miR‐669a‐5p suppresses G3BP expression to regulate cilium elongation.

### 2.4 Loss of G3BPs Leads to Elongated Cilia by Regulating AKAP12

**Figure 4:**
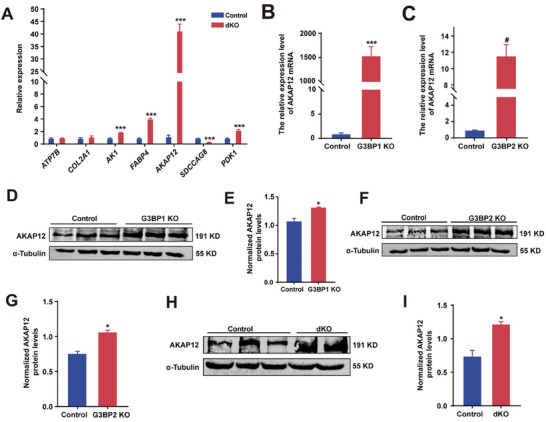
G3BPs regulate AKAP12 expression. A) qPCR analysis of differentially expressed genes in control and G3BP dKO cells. *n* = 4 experiments. B,C) Transcript levels of *AKAP12* were quantified by qPCR in control and *G3BP*1 KO cells (B), or control and G3BP2 KO cells (C). *n* = 4 experiments. D–I) Western blot of control and G3BP1 KO (D–E), or control and G3BP2 KO (F–G), or control and G3BP dKO (H–I) cell lysates probing with anti‐AKAP12. α‐Tubulin was used as a loading control. *n* = 3 experiments. All data were presented as mean ± SEM; ^*^ *p* < 0.05, ^***^ *p* < 0.001 and #*p* < 0.0001 by Student's *t*‐test or one‐way ANOVA and Bonferroni pairwise comparisons.

To explore how G3BPs regulate cilium length, we performed mass spectrometry to elucidate the protein expression profiles of control cells and G3BP dKO cells after culture in serum‐free medium for 24 h. A total of 105 differentially expressed proteins were identified, including 60 proteins that were significantly upregulated and 45 that were downregulated in G3BP dKO cells relative to control cells (Figure S4A and Table S3, Supporting Information). Among these differentially expressed proteins, we found seven cilium‐related proteins, namely, copper‐transporting ATPase 2 (ATP7B),^[^ ^24^ ^]^ collagen alpha‐1 (II) chain (COL2A1),^[^ ^25^ ^]^ aspartokinase 1 (AK1),^[^ ^26^ ^]^ fatty acid‐binding protein (FABP4),^[^ ^27^ ^]^ AKAP12,^[^ ^28^ ^]^ serologically defined colon cancer antigen 8 homolog (SDCCAG8),^[^ ^29^ ^]^ and pyruvate dehydrogenase (acetyl‐transferring) kinase isozyme 1 (PDK1) (Figure S4B and Table S4, Supporting Information). First, qPCR results showed that the transcript level of *SDCCAG8* was downregulated in G3BP dKO cells relative to control cells, which was inconsistent with the mass spectrometry results and that the levels of *ATP7B* and *CoL2A1* were indistinguishable between the two cell lines. Consistent with the mass spectrometry results, the expression levels of *AK1*, *AKAP12*, *FABP4*, and *PDK1* were upregulated in G3BP dKO cells, and *AKAP12* was the gene that was most highly expressed and exhibited the most significant upregulation (**Figure** **4**A). Moreover, *AKAP12* was markedly upregulated in G3BP1 KO or G3BP2 KO cells relative to control cells (Figure 4B,C). AKAP12 belongs to the A‐kinase‐anchored protein (AKAP) family.^[^ ^30^ ^]^ It is an essential component of the cAMP‐PKA signaling pathway, anchoring PKA to specific subcellular structures.^[^ ^31^ ^]^ Interestingly, the Kyoto Encyclopedia of Genes and Genomes (KEGG) enrichment analysis of the differentially expressed proteins suggested that G3BPs were involved in the MAPK and cAMP signaling pathways (Figure S4C, Supporting Information). These data suggest that G3BPs and AKAP12 may function in the same pathway. Furthermore, western blot analysis showed that AKAP12 expression was significantly increased in G3BP1 KO, G3BP2 KO, and G3BP dKO cells compared to control cells (Figure 4D–I). Thus, it is reasonable to hypothesize that G3BPs may regulate cilium length through AKAP12.

### 2.5 AKAP12 Deletion Reverses G3BP Deletion‐Mediated Cilium Elongation

**Figure 5:**
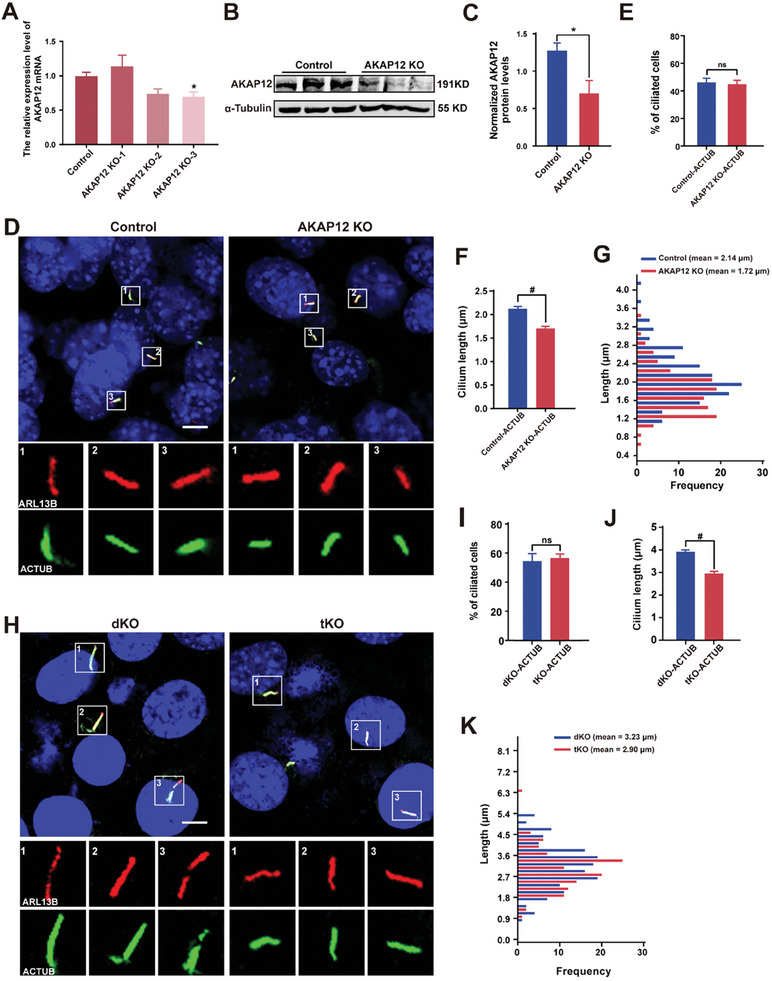
Ablation of AKAP12 reverses longer cilia upon G3BP deletion. A) AKAP12 KO cells were generated using CRISPR/Cas9 strategy by transient transfection with PX459 plasmids containing *AKAP12* gRNA (gRNA‐1, gRNA‐2, or gRNA‐3), and *AKAP12* levels were quantified by qPCR. *n* = 4 experiments. B,C) Western blot probing with anti‐AKAP12 in control and AKAP12 KO cells. α‐Tubulin was used as a loading control. *n* = 3 experiments. D–G) Control and AKAP12 KO cells were starved for 24 h and stained with anti‐ARL13B (red) and anti‐ACTUB (green) antibodies. DAPI (blue) was used to stain nuclei. Scale bar, 5 µm (D). Percentage of ciliated cells in control and AKAP12 KO cells (E). Quantification of cilium length in control and AKAP12 KO cells (F–G). H–K) G3BP dKO and tKO cells were starved for 24 h and stained with anti‐ARL13B (red) and anti‐ACTUB (green) antibodies. DAPI (blue) was used to stain nuclei. Scale bar, 5 µm (H). Percentage of ciliated cells in G3BP dKO and tKO cells (I). Quantification of cilium length in G3BP dKO and tKO cells (J,K). *n* = 3, a minimum of 150 cells were used for each group. All data represent the mean ± SEM; ns, not significant, ^*^ *p* < 0.05 and ^#^ *p* < 0.0001 by Student's *t*‐test or one‐way ANOVA and Bonferroni pairwise comparisons.

To investigate whether AKAP12 is involved in ciliogenesis, we used CRISPR/Cas9 technology to establish an AKAP12 knockout cell line. qPCR and western blot revealed that AKAP12 expression levels were downregulated in transiently transfected AKAP12 KO cells compared to control cells (**Figure** **5**A–C).

We then analyzed ciliogenesis in NC cells and AKAP12 KO cells. We found that the cilium formation of AKAP12 KO cells was not significantly different from that of control cells; however, the cilium length of AKAP12 KO cells was significantly shorter than that of control cells (Figure 5D–G). This result indicates that loss of AKAP12 impairs cilium elongation, which mirrors the result that ablation of G3BPs increased AKAP12 expression and promoted cilium elongation (Figure 3).

To define the relationship between G3BPs and AKAP12 in regulating cilium elongation, we examined cilium formation and length in G3BP‐AKAP12 triple KO (tKO) cells. Compared to G3BP dKO cells, tKO cells exhibited similar cilium formation but decreased cilium length (Figure 5H–K). Notably, the cilia of the tKO cells overall were still longer than those of control cells (2.14 ± 0.05 µm). Part of the reason lies in the transient transfection of the AKAP12 gRNA construct. Nevertheless, deletion of AKAP12 in the G3BP dKO context significantly reversed the cilium elongation that was caused by G3BP deletion, suggesting that G3BPs regulate cilium length by modulating AKAP12 expression.

### 2.6 Ablation of AKAP12 Reverses miR‐669a‐5p Mimic‐Mediated Cilium Elongation

**Figure 6:**
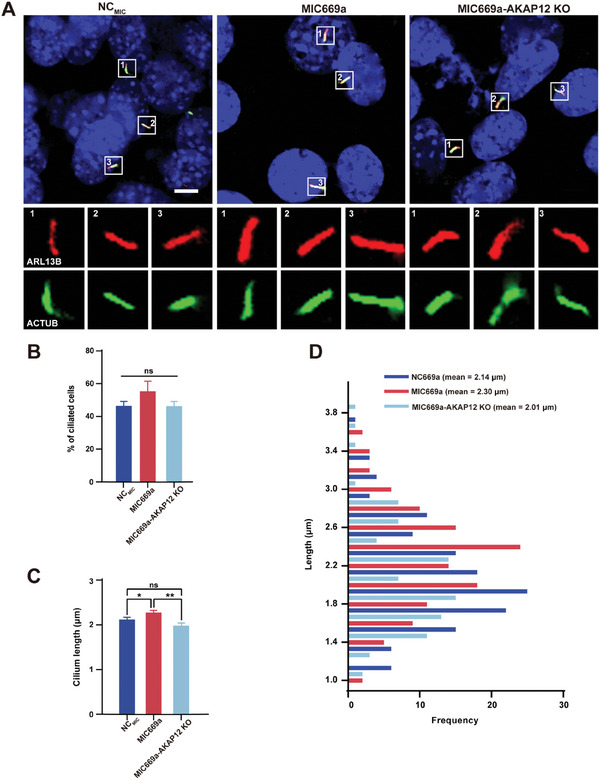
Cilium elongation promoted by miR‐669a‐5p mimics is reversed by AKAP12 deletion. A) AKAP12 KO cells treated with NC_MIC_ or MIC669a were starved for 24 h followed by staining with anti‐ARL13B (red) and anti‐ACTUB (green) antibodies. DAPI (blue) was used to stain nuclei. Scale bar, 5 µm. B) Percentage of ciliated cells in NC_MIC_, MIC669a, and MIC669a‐AKAP12 KO cells. C,D) Quantification of cilium length in NC_MIC_, MIC669a, or MIC669a‐AKAP12 KO cells. *n* = 3, a minimum of 150 cells were used for each group. All data were presented as mean ± SEM; ns, not significant, ^*^ *p* < 0.05 and ^**^ *p* < 0.01 by Student's *t*‐test or one‐way ANOVA and Bonferroni pairwise comparisons.

Having demonstrated that cilium length was regulated by miR‐669a‐5p/G3BPs and G3BPs/AKAP12, we reasoned that miR‐669a‐5p targets G3BPs, which leads to AKAP12 upregulation and ultimately cilium elongation. To test this hypothesis, we investigated whether miR‐669a‐5p mimic‐induced cilium elongation could be reversed to control levels by deleting AKAP12. NIH3T3 control or AKAP12 KO cells were transfected with miR‐669a‐5p mimics, and cells that were transfected with miR‐669a‐5p NC were used as control. Cilium formation was not significantly different between the treated and control groups (**Figure** **6**A,B). In contrast, the cilium length of the miR‐669a‐5p mimic‐treated AKAP12 KO group (2.01 ± 0.06 µm) was significantly shorter than that of the miR‐669a‐5p mimic‐treated group (2.30 ± 0.05 µm), and more importantly, it was similar to that of the NC group (2.14 ± 0.05 µm) (Figure 6C,D). Collectively, these results indicate that cilium elongation caused by miR‐669a‐5p overexpression is counteracted by inhibition of AKAP12 expression, providing compelling evidence that miR‐669a‐5p regulates cilium length through the G3BP/AKAP12 axis.

### 2.7 HDAC6 Mediates G3BP Inhibition of AKAP12 to Suppress Cilium Disassembly

**Figure 7:**
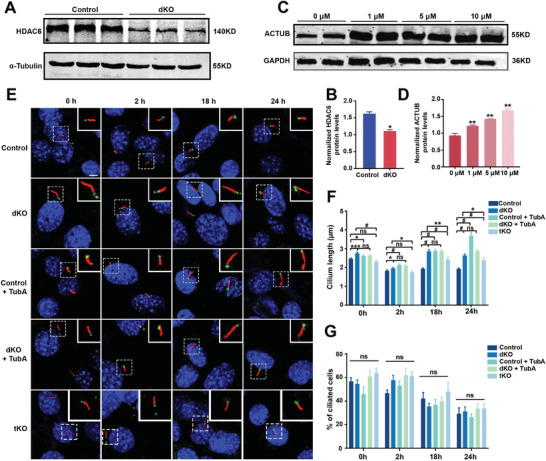
HDAC6 mediates G3BPs’ inhibition on AKAP12 to suppress cilium disassembly. A) Western blot probing with anti‐HDAC6 in control and G3BP dKO cells. α‐Tubulin was used as a loading control. B) Quantification of HDAC6 expression levels in control and dKO cells. *n* = 3 experiments. C) Western blot probing with anti‐ACTUB in cells treated with TubA at 0, 1, 5, and 10 μm. GAPDH was used as a loading control. D) Quantification of ACTUB expression levels in TubA‐treated cells. *n* = 3 experiments. E,F) Cells were starved in serum‐free medium for 24 h followed by serum stimulation for 0, 2, 18, and 24 h and were stained with anti‐ARL13B (red) and anti‐γ‐Tubulin (green) antibodies. DAPI (blue) was used to stain nuclei. Scale bar, 5 µm, (E). Quantification of cilium length in control, dKO, control + TubA, dKO + TubA, and tKO cells (F). *n* = 3, a minimum of 150 cells were used for each group. G) Percentage of ciliated cells in control, dKO, control + TubA, dKO + TubA, and tKO cells. *n* = 3, a minimum of 150 cells were used for each group. All data were presented as mean ± SEM; ns, not significant, ^*^ *p* < 0.05, ^**^ *p* < 0.01 and #*p* < 0.0001 by Student's *t*‐test or one‐way ANOVA and Bonferroni pairwise comparisons.

How G3BPs regulate AKAP12 expression is unknown. Interestingly, HDAC6 interacts with G3BPs in stress granules, and HDAC6 associates with AKAP12 in cancer cells.^[^ ^32^ ^]^ We therefore speculated that G3BPs might regulate AKAP12 expression via HDAC6. Western blot analysis revealed that HDAC6 expression was significantly downregulated in G3BP dKO cells compared to control cells (**Figure** **7**A,B), indicating that HDAC6 expression is repressed in the absence of G3BPs. To further explore the role of HDAC6, we overexpressed HDAC6 in G3BP dKO cells and found that overexpression of HDAC6 effectively inhibited AKAP12 expression (Figure S5A–C, Supporting Information). Collectively, our results highlighted that HDAC6 mediates the regulation of AKAP12 expression by G3BPs.

How does miR‐669a‐5p regulate cilium length? Our results described above showed that HDAC6 links G3BPs to AKAP12. Previous results showed that HDAC6 mainly functions in cilium disassembly.^[^ ^33^ ^]^ Therefore, we reasoned that miR‐669a‐5p regulates cilium length primarily by affecting cilium disassembly by targeting G3BPs. To test this hypothesis, we used tubastatin A (TubA), a specific inhibitor of HDAC6, to treat control or G3BP dKO cells.^[^ ^34^ ^]^ TubA significantly inhibited HDAC6 expression in a dose‐dependent manner. Accordingly, we chose an intermediate dose (5 µM) in the subsequent experiments (Figure 7C,D). Reportedly, cilia disassembly occurs in two waves after serum stimulation, with one occurring after 2 h of serum treatment and another after 18–24 h. Thus, we starved cells in serum‐free medium for 24 h and then added serum and incubated the cells for 0, 2, 18, and 24 h. IFA showed that the cilia of G3BP dKO cells were significantly longer than those of control cells at all time points, suggesting that the cilia of G3BP dKO cells were resistant to serum‐induced cilia disassembly (Figure 7E,F). Moreover, the addition of TubA markedly inhibited cilium disassembly in control cells but not in G3BP dKO cells, indicating that, without G3BPs, the cells were not able to respond to TubA (Figure 7E,F). In addition, compared to G3BP dKO cells, tKO cells also exhibited shorter cilia and restored kinetics of cilium disassembly (Figure 7E,F). Notably, cilium formation in G3BP dKO and tKO cells tended to decrease in the presence of TubA, but the difference was not significant (Figure 7G). Altogether, these results demonstrate that miR‐669a‐5p affects cilium length mainly by regulating cilium disassembly.

## 3 Discussion

In this study, we revealed a mechanism by which a key miRNA, namely, miR‐669a‐5p, regulates cilium length. We demonstrate that miR‐669a‐5p targets G3BPs to regulate the expression of HDAC6 and then AKAP12, thereby increasing cilium length by preventing cilium disassembly. Our work correlates miR‐669a‐5p with the regulation of cilium length, providing insights into the etiology of ciliopathies and revealing implications for treatment.

miR‐669a‐5p, which is transcribed from the 10th intron of the *Sfmbt2* gene, has been implicated in various diseases.^[^ ^18b^ ^]^ For instance, miR‐669a‐5p is enriched in adipocytes and contributes to the “browning” of adipose tissues.^[^ ^35^ ^]^ Interestingly, the differentiation and accumulation of adipocytes are related to the transient formation of cilia. Adipocytes that are cultured from dermal fibroblasts of patients with Bardet‐Biedl syndrome exhibit higher levels of fat accumulation and leptin secretion.^[^ ^36^ ^]^ Moreover, miR‐669a is significantly enriched in embryonic stem cells, maintaining stem cell pluripotency and self‐renewal.^[^ ^37^ ^]^ During the differentiation of cardiac progenitor cells, miR‐669a can prevent skeletal myogenesis by targeting MyoD.^[^ ^38^ ^]^ In addition, sustained overexpression of miR‐669a‐5p relieves the symptoms of muscular dystrophy and improves the survival of mice with severe cardiomyopathy.^[^ ^39^ ^]^ However, how miR‐669a‐5p regulates the progression of these diseases remains largely unexplored. Given the important role of miR‐669a‐5p in disease development, we reason that it is highly possible that miR‐669a‐5p is involved in the regulation of cilium length, and elucidation of the pertinent mechanism will facilitate ciliopathy treatment.

Upon encountering harsh environments such as oxidative stress and nutrient deficiency, eukaryotes initiate a cascade of responses to form stress granules as a major mechanism of cell protection.^[^ ^40^ ^]^ Stress granules are organelles that lack membranes and are rich in mRNP granules. G3BPs, which are the core components of stress granules, play essential roles in cellular responses to various stresses.^[^ ^41^ ^]^ G3BPs can anchor the tuberous sclerosis complex (TSC) to lysosomes and inhibit rapamycin complex 1 (mTORC1) activation by amino acids and insulin.^[^ ^40a^ ^]^ The elevated expression of mTORC1 in TSC patients and mutant mice results in fewer cilia.^[^ ^42^ ^]^ In addition, changes in cilia length occur in time frames ranging from minutes to hours, and such changes often occur due to stress.^[^ ^3^ ^]^ However, the function of G3BPs in cilium development in vertebrates is unknown. Our data indicate that G3BPs link miR‐669a‐5p with downstream target proteins, significantly contributing to the regulation of cilium length. The demonstrated role of G3BPs in the regulation of cilium length not only broadens the function of G3BPs but also expands our understanding of how cilium length is regulated.

To elucidate the role of G3BPs in regulating cilium length, we constructed a G3BP double knockout cell line and performed proteomic analysis. We found that knocking out G3BPs significantly increased the expression level of AKAP12. AKAP12, which is a member of the AKAP family, was originally identified in the serum of myasthenia gravis patients. It is an integral component of the cytoskeleton, controlling mitotic signal transduction and cytoskeletal remodeling by binding to key signaling components such as protein kinase A (PKA), protein kinase C (PKC), and cyclins.^[^ ^43^ ^]^ AKAP12 is a negative regulator of the G1/S phase transition, and its high expression can block Cyclin‐D1 synthesis and concurrently prolong the G1 phase, suggesting that AKAP12 is involved in ciliogenesis, which occurs during the G1/G0 transition. Consistent with our results, previous studies have implicated several other AKAP family members in regulating cilium development and functions. For example, G‐protein coupled receptor 161 is localized to cilia and antagonizes Hh signaling by activating PKA in the absence of Hh ligands;^[^ ^44^ ^]^ mutation of AKAP450 disrupts the connection between the Golgi apparatus and the centrosome, thus preventing ciliogenesis.^[^ ^45^ ^]^ Collectively, these results highlight the role of AKAP family members, particularly AKAP12, in the regulation of cilium length.

Then, how do G3BPs regulate AKAP12? Reportedly, G3BPs interact with HDAC6, which is a core component of stress granules, and AKAP12 binds to HDAC6 in human colon cancer cells.^[^ ^32^ ^]^ Our results demonstrated that HDAC6 was significantly downregulated in G3BP dKO cells, suggesting that G3BPs can modulate HDAC6 expression. Moreover, overexpression of HDAC6 significantly decreased the abundance of AKAP12 in G3BP dKO cells, indicating that HDAC6 negatively regulates AKAP12 expression. HDAC6 is a unique cytoplasmic enzyme that is composed of two functionally homologous catalytic domains and a ubiquitin‐binding zinc finger domain in the carboxy‐terminal region; HDAC6 regulates various cellular signaling pathways through its deacetylase and ubiquitin‐binding activities.^[^ ^46^ ^]^ Furthermore, phosphorylation of G3BPs determines the binding of G3BPs to HDAC6, and the ubiquitin and deacetylation domains of HDAC6 are essential for granule cell formation. AKAP12 is a substrate of HDAC6 deacetylation and is tightly regulated by HDAC6. Based on this, we believe that HDAC6 mediates the regulation of AKAP12 by G3BPs.

AKAP12 forms a macromolecular complex with Aurora A and Polo‐like Kinase 1 (PlK1) during mitosis. Whether AKAP12 directly affects cilium length is unknown, and the relevant molecular mechanism has not yet been elucidated.^[^ ^28^, ^47^ ^]^ Accumulating evidence indicates that dysregulation of HDAC6 contributes to the pathogenesis of ciliopathies, and its activation requires phosphorylation by Aurora A and PlK1. Activated HDAC6 subsequently stimulates AC‐α‐Tubulin deacetylation, destabilizes axoneme microtubules, and induces cilium resorption to drive cilium disassembly.^[^ ^33^, ^48^ ^]^ Our results demonstrate that miR‐669a‐5p regulates cilium elongation through the G3BP/HDAC6/AKAP12 axis and that HDAC6 links G3BPs with AKAP12. Therefore, it is most likely that miR‐669a‐5p regulates cilium length mainly by affecting cilium disassembly. Reportedly, the shortened cilia phenotype can be partially reversed by treating cells with Aurora A or HDAC6 inhibitors.^[^ ^49^ ^]^ Our study shows that loss of G3BPs prevents cilium disassembly, promoting ciliu elongation. However, overexpression of HDAC6 or deletion of AKAP12 in G3BP‐deficient cells partially restored cilium disassembly. In addition, overexpression of HDAC6 significantly decreased the expression level of AKAP12 in G3BP‐deficient cells. Taken together, these suggest an important regulatory role of the miR‐669a‐5p/G3BP/HDAC6/AKAP12 axis in cilium disassembly.

Reportedly, miRNA regulates gene expression by binding to mRNAs. How does miR‐669a‐5p, which is enriched in cilia enriched noncellular fraction, regulate the expression of G3BPs in cells? We speculate that the miR‐669a‐5p/G3BPs/HDAC6/AKAP12 axis may exploit the extracellular vesicle (EV) pathway to regulate cilium length for the following reasons. EVs are secretory vesicles that can carry mRNA, miRNA, DNA, and proteins,^[^ ^50^ ^]^ and evidence has already shown that cilia are specialized organelles responsible for EV biogenesis and reception.^[^ ^51^ ^]^ Reportedly, in morphine‐treated astrocytes, EVs containing miR‐106b can promote ciliogenesis in cells by downregulating CEP97.^[^ ^52^ ^]^ Furthermore, in human β‐cells, colocalization of G3BP1‐RFP and CD63‐GFP (EV marker) indicates that stress granules and EVs are present together, and the contents of stress granules can be incorporated into EVs.^[^ ^53^ ^]^ Therefore, G3BPs and HDAC6, two key components of stress granules, can be readily recruited by EVs. As such, miR‐669a‐5p, G3BPs, and HDAC6 can simultaneously appear in EVs that are excreted from cells through exocytosis and then fuse with the ciliary membrane to deliver their contents to cilia. Additionally, HDAC6 is located at the base of cilia and even throughout the entire cilia.^[^ ^54^ ^]^ Hence, HDAC6 and AKAP12 may also directly pass through the ciliary transition zone to interact with G3BPs that are released from EVs. However, how miR‐669a‐5p and G3BPs are transported by EVs merits further investigation.

Previous studies have shown that most ciliopathy‐related miRNAs can affect both cilium formation and length. In this study, miR‐669a‐5p overexpression did not affect cilium formation but acted as a potent regulator of cilium length by modulating cilium disassembly. Currently available drugs, such as inhibitors that target HDAC6 or Aurora A, simultaneously affect cilium formation and disassembly.^[^ ^48a^ ^]^ Therefore, they are not suitable for treating diseases that are specifically caused by abnormal cilium length. Here, our study identified miR‐669a‐5p as a specific regulator of cilium length, providing a possibility for precision therapy to manipulate miR‐669a‐5p expression levels.

In summary, our study reveals a novel mechanism by which cilium length is regulated. miR‐669a‐5p targets G3BPs to inhibit HDAC6 expression and thus to increase AKAP12 expression, thereby preventing cilium disassembly and promoting cilium elongation. Our findings provide valuable evidence to improve the understanding of how cells maintain normal cilium length and may provide new therapeutic targets for treating ciliopathies.

## 4 Experimental Section

### Cell Culture, Plasmids, and Transfection

Mouse fibroblasts (NIH3T3) cells were purchased from Pricella (Wuhan, China) and cultured in DMEM (Gibco) supplemented with 10% fetal bovine serum (FBS) and 1% penicillin/streptomycin (Gibco). NIH3T3 cells were starved in DMEM supplemented with 0.5% FBS and 1% penicillin/streptomycin for 24 h to induce cilium growth. Guide RNAs (gRNAs) were designed to target the region close to the start codon, preventing creation of truncated proteins. Oligonucleotides for gRNA cloning were listed in Table S5 (Supporting Information). Briefly, the gRNA was cloned into the BbsI sites of pSpCas9 (BB)−2A‐Puro vector (pX459, #48139, Addgene) and was validated by Sanger sequencing. pcDNA3.1‐HDAC6 overexpression vector (C05008) was purchased from Genepharma (Shanghai, China). miR‐669a‐5p‐sponges inhibitor vector was constructed by GENE (Shanghai, China). NIH3T3 cells were transfected with respective plasmids using Lipofectamine 3000 according to the manufacturer's instructions.

### RNA Oligonucleotides and Transfection

miRNA Inhibitor and Mimics were obtained from Gemma (Suzhou, China), with the following sequences: miRNA NC from Gemma (B04003); miR‐669a‐5p mimics, 5′‐AGUUGUGUGUGCAUGUUCAUGUCU‐3′; miR‐669a‐5p inhibitors, 5′‐AGACAUGAACAUGCACACACAACU‐3′; miR‐7a‐5p mimics, 5′‐UGGAAGACUAGUGAUUUUGUUGU‐3′; miR‐7a‐5p inhibitors, 5′‐ACAACAAAAUCACUAGUCUUCCA‐3′; miR‐669o‐5p mimics, 5′‐UAGUUGUGUGUGCAUGUUUAUGU‐3′; miR‐669o‐5p inhibitors, 5′‐ACAUAAACAUGCACACACAACUA‐3′; miR‐466c‐5p mimics, 5′‐UGAUGUGUGUGUGCAUGUACAUAU‐3′; miR‐466c‐5p inhibitors, 5′‐AUAUGUACAUGCACACACACAUCA‐3′. NIH3T3 cells at 60–70% confluency were transfected with miRNA NC, mimics, inhibitors, or siRNA using Lipofectamine 3000 Reagent (L3000015, Invitrogen) according to the manufacturer's instructions.

### Isolation of Noncellular Fraction by Calcium‐Shock Method

The calcium‐shock method was adopted from previously described literatures with slight modifications.^[^ ^55^ ^]^ Briefly, NIH3T3 cells were cultured in 70×10 cm dishes. After 24 h of starvation, the cells were treated with 0.04% EDTA for 10 min, and washed with HEPES‐buffered saline. The cell pellets were resuspended in deciliation solution with 10 µg ml^−1^ Cytochalasin D (C102396, Aladdin) plus 1 U/µl RNase Inhibitor (2313A, TaKaRa). Subsequently, samples were purified by ultracentrifugation (Hitachi) using 30–45% (w/w) discontinuous and 30–45% (w/w) continuous sucrose gradients. More than five times of HE solution was used to wash the samples. The precipitate was resuspended in 30 µL of HE solution followed by vortex for 10 min at 4 °C and was stored at −80 °C.

### Small RNA Sequencing Analyses

Small RNA sequencing of the noncellular fraction (*n* = 2 experiments) and cellular fraction (*n* = 2 experiments) from NIH3T3 cells by Novogene (Beijing, China). Sequencing libraries were generated using a NEBNext Multiplex Small RNA Library Prep Set for Illumina (NEB) following the provided protocol. The library preparations were sequenced on an Illumina HiSeq 2500/2000 platform, and 50 bp single‐end reads were generated. The original data (raw reads) were processed through custom Perl and Python scripts. Differential expression analysis was performed using the DESeq R package (1.8.3).

### Luciferase Assay

The TargetScan (http://targetscan.org) and miRWalk (Home – miRWalk (uni‐heidelberg.de) were used to identify the putative target genes of miR‐669a‐5p. The sequence of mouse *G3BP1* (NM_013716; ENSMUSG00000018583.5) and *G3BP2* (NM_001080794; ENSMUSG00000029405.16) were obtained from the GenBank and UCSC Genome Browser. Information on miR‐669a‐5p (MI0004523) was acquired from miRBase.

To construct the luciferase reporters, a part of *G3BP1* CDS or 3′UTR and *G3BP2* 3′UTR containing the miR‐669a‐5p potential target sites was cloned in the pmirGLO Dual‐Luciferase Vector (E1330, Promega). The Mut Express II Fast Mutagenesis Kit V2 (C214‐01, Vazyme) was used for target sequence mutagenesis. The primers for constructing plasmids were listed in Table S5 (Supporting Information). Briefly, cells were co‐transfected with the dual‐luciferase plasmid containing *G3BP1* CDS or 3′UTR, *G3BP2* 3′UTR, or the respective mutated sequences, miR‐669a‐5p mimic (MIC669a), or nontargeting NC (NC_MIC_) oligos and were them cultured for 48 h before the luciferase assay. Firefly and Renilla luciferase activities were detected with a dual‐luciferase reporter assay system (E2940, Promega), and the results were shown as firefly luciferase activity relative to Renilla luciferase activity.

### RNA Isolation and qPCR Quantification

Total miRNAs were extracted using the miRNeasy Mini Kit (217004, QIAGEN), and reverse transcription was performed using a Mir‐X miRNA First‐Strand Synthesis Kit (638313, TaKaRa). Expression of mature miR‐669a‐5p was detected using Nova SYBR PCR Master Green mix (63029181, QIAGEN) and miR‐669a‐5p qPCR primers. Total RNA was isolated using the RNeasy Micro Kit (217084, QIAGEN), and first‐strand cDNA was synthesized using a PrimeScipt RT reagent Kit with gDNA eraser (R323‐01, Vazyme). The qPCR was performed with Nova SYBR Green PCR Master Green mix (R712, Vazyme). Gene expressions were normalized to *GAPDH* levels and were determined by the 2^−ΔΔCT^ method.^[^ ^56^ ^]^ Primer sequences used for qPCR were listed in Table S5 (Supporting Information).

### LC‐MS/MS and Bioinformatic Analysis

Cells (*n* = 3 experiments) were collected and the total proteins were extracted with Sodium deoxycholate (DOC) lysis buffer (50 mm NH_4_HCO_3_, 2% Sodium deoxycholate, 25 mm NaCl, pH = 8.5) supplemented with protease inhibitors. A total of 100 µg protein was resuspended in 5 mm dithiothreitol at 95 °C for 3 min, and were digested by 2 µg trypsin (V5280, Promega) for 6 h, followed by treatment with 2 µg trypsin for 14 h at 37 °C. The digested peptides were desalted using a Sep‐Pak C18 column (A57003100, Agilent Technologies). The peptides were then lyophilized, reconstituted in 0.1% formic acid, and analyzed using a Q Exactive Mass Spectrometer (Thermo Fisher Scientific) coupled to an UltiMate 3000 RSLC Systems (Thermo Fisher Scientific). The spectral data were analyzed using the Proteome Discoverer software (Thermo Fisher Scientific, version 2.2) by searching against the mouse proteins in the UniProt database.

### Western Blot Assays

Cells were lysed in lysis buffer (P0013, Beyotime) with a protease inhibitor cocktail (100 ×) (5871, Cell Singling Technology) for 20 min on ice, followed by centrifugation at 12000 × g for 20 min at 4 °C. Proteins were separated by SDS–PAGE and transferred to a PVDF membrane (RINB77899, Millipore). The membrane was blocked for 1 h in TBS plus 5% nonfat milk, followed by incubation at 4°C overnight or at room temperature (RT) for 2–3 h with primary antibodies. After three washes with TBST, the membrane was incubated with secondary antibodies at RT for 1 to 2 h. Images were obtained using Odyssey software (Gene). Antibodies used in this study include: anti‐G3BP1 (1:1000; ab181150, Abcam), rabbit anti‐G3BP2 (1:1000; HPA018425, Sigma), rabbit anti‐AKAP12 (1:1000; ER1903‐55, HUABIO), GAPDH (1:1000; HC301‐01, TransGen Biotech); rabbit anti‐HDAC6 (1:1000; 6712S, Cell Signaling Technology). Secondary antibody: conjugated with 800 nm fluorophores (SeraCare KPL, 1:10000).

### Immunofluorescence and Image Analysis

Cells were grown on coverslips in 24‐well plates and then fixed with 4% polyformaldehyde (PFA) in PBS at RT for 15 min. After two washes with PBS, cells were permeabilized by 0.2% Triton X‐100 in PBS for 20 min. After blocking in 10% goat serum in PBS, the cells were incubated with primary antibodies overnight at 4°C. The next day, the cells were washed three times with PBS, incubated with Alexa Fluor‐conjugated secondary antibodies for 2 h, and then mounted with an anti‐quenching sealing agent (0100‐01, Southern Biotech). Finally, cell images were taken using an Olympus FLUOVIEW FV3000 confocal microscope with a 60 × NA 1.4 Pan Apo oil immersion objective (Olympus) and processed with FV10‐ASW software (Olympus). Primary antibodies used include rabbit anti‐ARL13B (1:1000; 17711‐1‐AP, Proteintech) and mouse anti‐acetylated tubulin (1:2000; T6793, Sigma). Secondary antibodies: Alexa 488‐ and 594‐ conjugated antibodies (1:1000; Thermo Scientific).

### Cell Cycle Analysis

The cell cycle was determined by FACS analysis using 1 µg ml^−1^ DAPI. Cells were transfected with NC and mimics, respectively. After 48 h, cells were washed with cold PBS twice and were fixed in ice‐cold 80% ethanol (vol/vol) overnight. The cells were pelleted at 1200 × g and resuspended in 0.5 ml of a freshly made 1 µg ml^−1^ DAPI solution at room temperature for 10 min. Data were analyzed and plotted using the ModFit LT 5.0 software.

### Statistical Analysis

Statistical analyses were performed in GraphPad Prism 8.0 using Student's two‐tailed *t*‐test or ANOVA. All data were presented as mean ± SEM. Differences were considered significant if *p* < 0.05 (^*^ *p* < 0.05, ^**^ *p* < 0.01, ^***^ *p* < 0.001, ^#^ *p* <0.0001).

## Conflict of Interest

The authors declare no conflict of interest.

## Author Contributions

W.W. and X.D. contributed equally to this work. W.W., X.D., X.H., and Z.W. conceptualized the idea for the study. W.W., X.D., Y.L., M.L., Q.C., X.H., and Z.W. performed Investigation, visualization, and analysis. W.W., X.D., X.H., and Z.W. wrote, reviewed, and edited the final manuscript. X.H. and Z.W. performed supervision. W.W., X.D., X.H., and Z.W. performed project administration and funding acquisition.

## Supporting information

Supporting Information

Supporting Information

Supporting Information

Supporting Information

## Data Availability

The data that support the findings of this study are available in the supplementary material of this article.
