# Supplementary material for: The miR‐669a‐5p/G3BP/HDAC6/AKAP12 Axis Regulates Primary Cilia Length
Source: Adv Sci (Weinh). 2023 Dec 13;11(6):2305068. doi: 10.1002/advs.202305068 (PMC10853727; doi:10.1002/advs.202305068)
Supplement: Supplementary file 1 — Supporting Information [file ADVS-11-2305068-s004.pdf]

ADVANCED SCIENCE
Open Access

Supporting Information

for *Adv. Sci.*, DOI 10.1002/advs.202305068

The miR-669a-5p/G3BP/HDAC6/AKAP12 Axis Regulates Primary Cilia Length

Weina Wang, Xuyao Dai, Yue Li, Mo Li, Zongqi Chi, Xiaoyu Hu and Zhenshan Wang**

Supporting Information

Title The miR-669a-5p/G3BP/HDAC6/AKAP12 axis regulates primary cilia length

Weina Wang[1]†, Xuyao Dai[1]†, Yue Li[1], Mo Li[2], Zongqi Chi[2], Xiaoyu Hu[1]*, and Zhenshan Wang[1]*

Content

Supplementary Figures (Figure S1-S5)

Supplementary Tables (Table S4-S5)

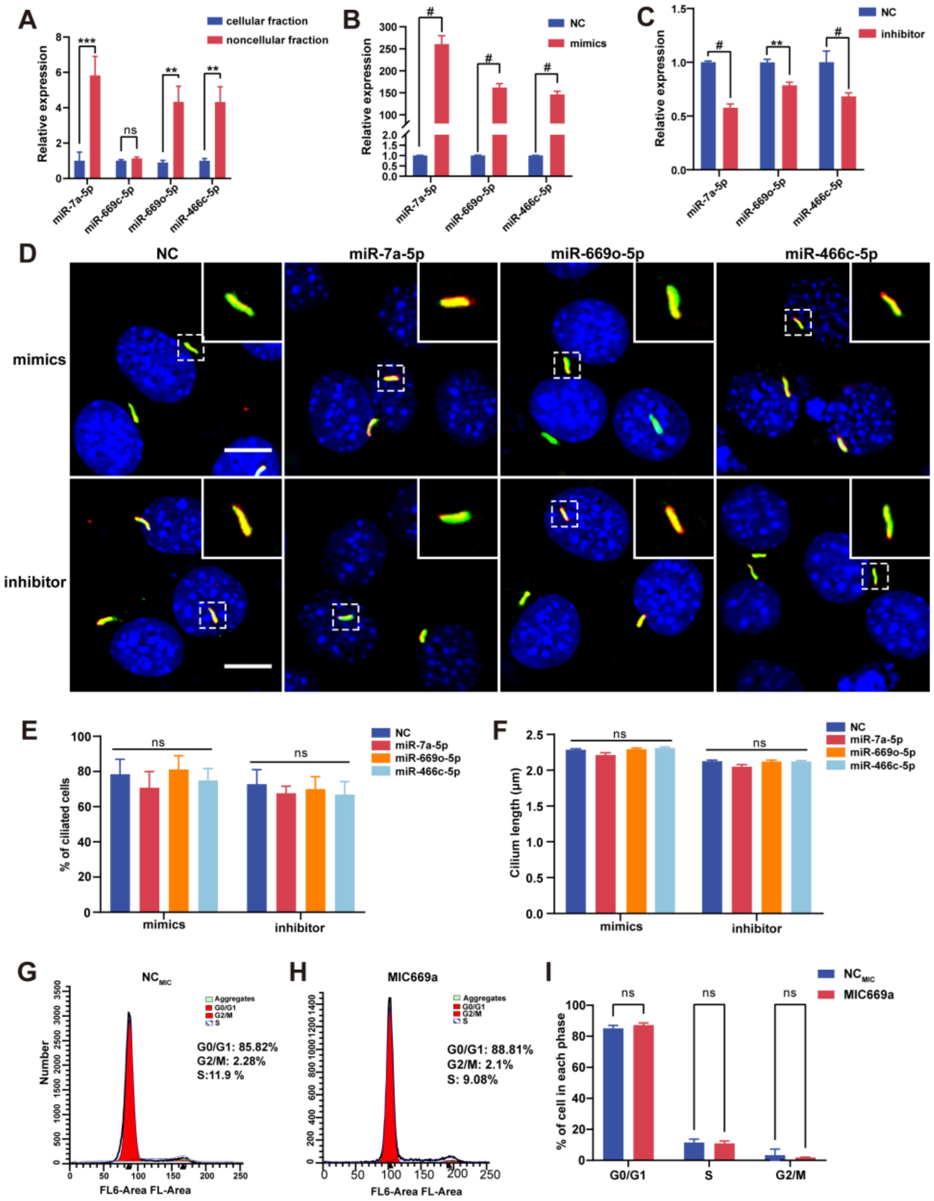

Supplementary Figure S1. Cilium length and formation are not affected by miR-7a-5p, miR-669c-5p, miR-669o-5p, and miR-466c-5p.

A. qPCR analysis of miR-7a-5p, miR-669c-5p, miR-669o-5p, and miR-466c-5p expression levels in the cellular and noncellular fractions, $n = 3$. **B-C.** qPCR analysis of miR-7a-5p, miR-669o-5p, and miR-466c-5p in cells treated with NC, mimics, and inhibitors, respectively, starved for 24 h. $n = 4$ from four independent experiments. **D.** Cells transfected with NC, mimics or inhibitors for miR-7a-5p, miR-669o-5p, and miR-466c-5p respectively were starved for 24 h followed by staining with anti-ARL13B (red) and anti-AC-α-Tubulin (green) antibodies. DAPI (blue) was used to stain nuclei. Scale bars, 5 µm. **E-F.** Percentage of ciliated cells in (D), and quantification of cilium length in (D). $n = 3$, a minimum of 150 cells per group were used for each group. **G-H.** Histograms showing cell cycle analysis through DAPI staining and flowing flow cytometry. Cells treated with NC_{MIC} or MIC669a were labeled for 48 h. $n = 3$ from four independent experiments. **I.** Quantification of cells in each stage shown in panel G and H. All data were represented as mean ± SEM; ns, not significant; $*p < 0.05$; $\#p < 0.0001$; Student's *t*- test or one-way ANOVA and Bonferroni pairwise comparisons.

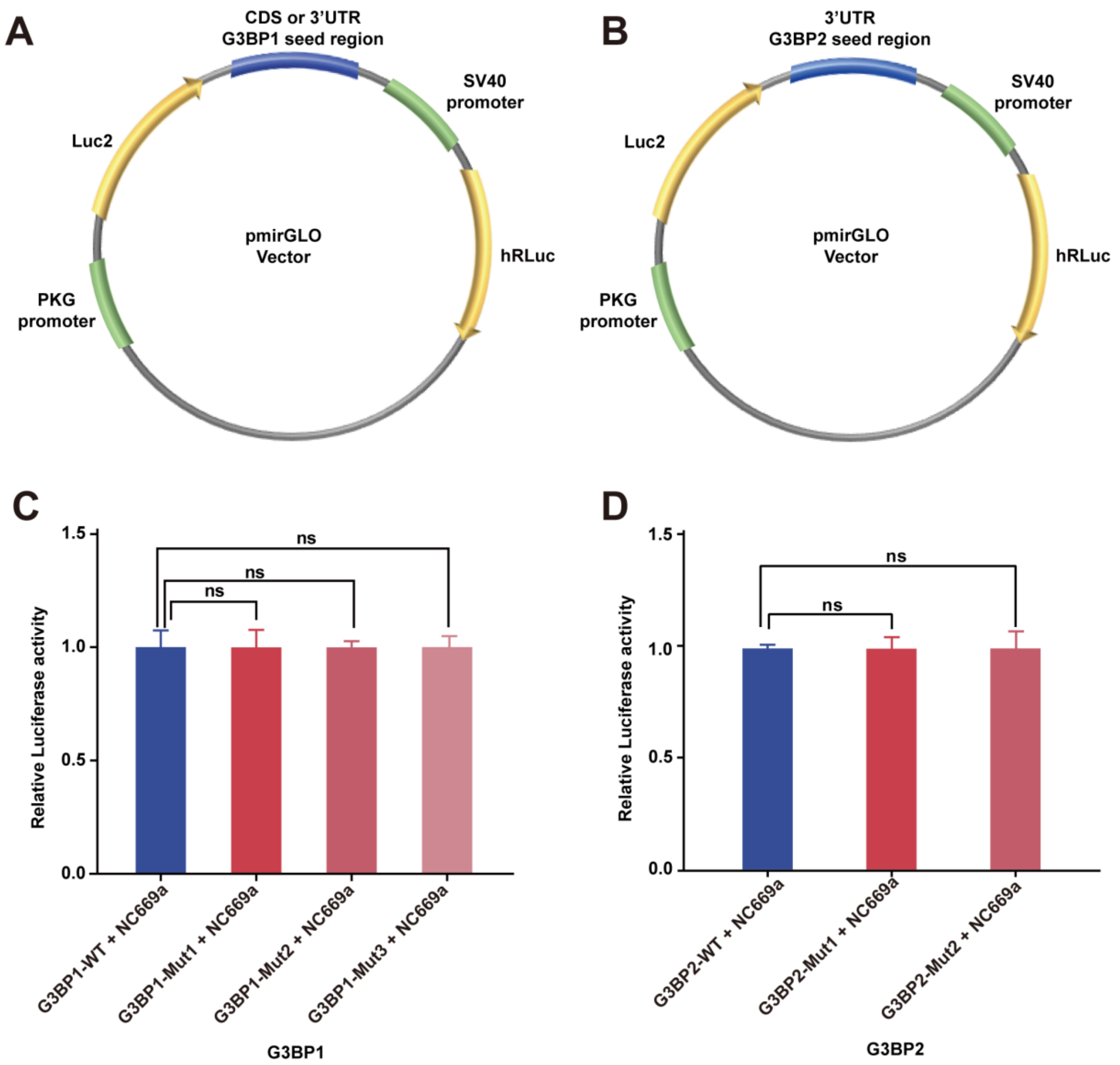

Supplementary Figure S2. G3BP1 and G3BP2 expressions are regulated by miR-669a-5p. **A-B.** Diagrams of the dual luciferase vectors containing *G3BP1* CDS or 3'UTR (A) and *G3BP2* 3'UTR (B). **C-D**. Luciferase activities were measured in cells transfected with *G3BP1* CDS, *G3BP2* 3'UTR, or respective mutations in luciferase vectors. All data were presented as mean ± SEM; Four replicates for each experiment. ns, not significant; One-way ANOVA and Bonferroni pairwise comparisons.

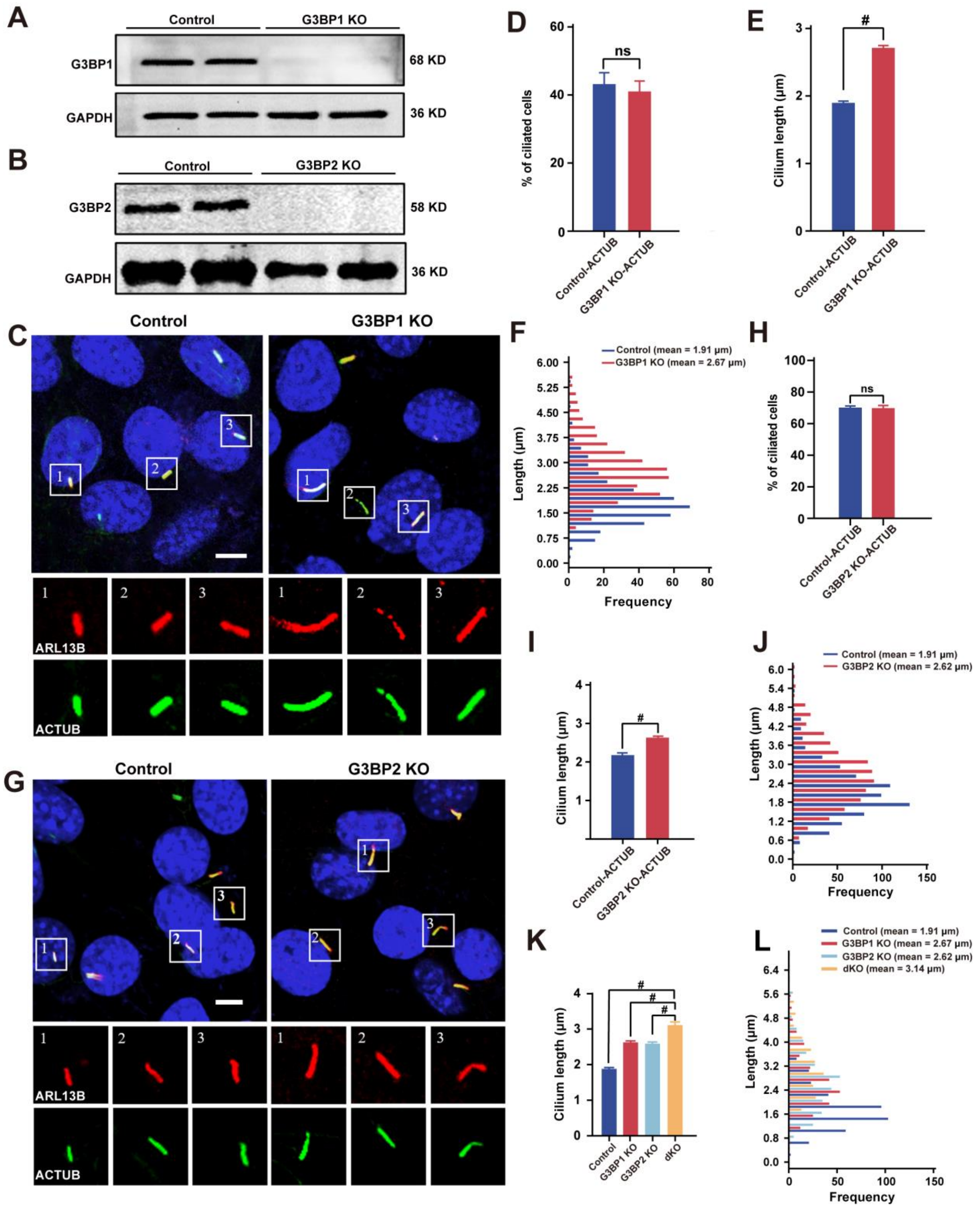

Supplementary Figure S3. Loss of G3BP1 or G3BP2 promotes cilium elongation.

A-B. Western blot of cell lysates of G3BP1 KO or G3BP2 KO cells probed with anti-G3BP1 or anti-G3BP2. GAPDH was used as a loading control. *n* = 3. **C, G.** Control, G3BP1 KO, and

G3BP2 KO cells were stained with anti-ARL13B (red) and anti-AC-α-Tubulin (green) antibodies following starvation for 24 h. DAPI (blue) was used to stain nuclei. Scale bars, 5 μm. **D-F, H-L.** Quantification of cells with primary cilia and cilium length of control, G3BP1 KO, G3BP2 KO, and dKO cells. $n = 3$, a minimum of 150 cells per group were used for each group. All data were presented as mean ± SEM; ns, not significant; #$p < 0.0001$; Student's *t*-test or one-way ANOVA and Bonferroni pairwise comparisons.

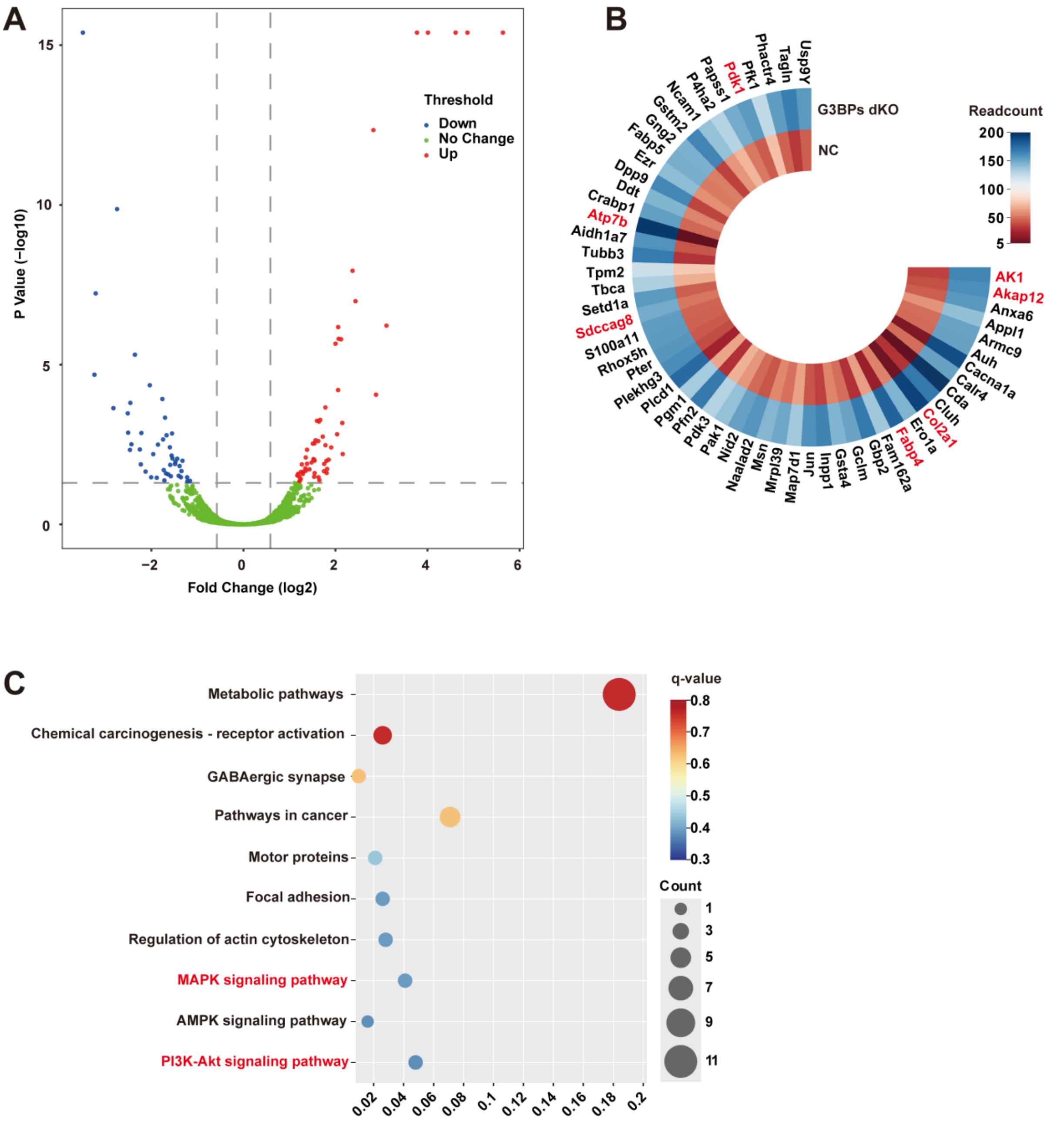

Supplementary Figure S4. LC-MS/MS analysis of G3BP-depleted cells.

A. Volcano plot of differentially expressed proteins between control and G3BP dKO cells. The upregulated proteins (> 2-fold-change, p < 0.05) and downregulated proteins (< 0.67-fold-change, p < 0.05) are highlighted in red and blue, respectively. Green spots indicate no significant change. **B.** Heatmaps showing enriched proteins among upregulated proteins in

G3BP dKO cells compared with the control cells revealed by LC-MS/MS. $n = 2$; fold change > 2. **C.** Kyoto Encyclopedia of Genes and Genomes (KEGG) pathway enrichment of upregulated genes in G3BP dKO cells. Dot size represents the number of genes enriched in the pathway. Dot color represents the significance of the difference.

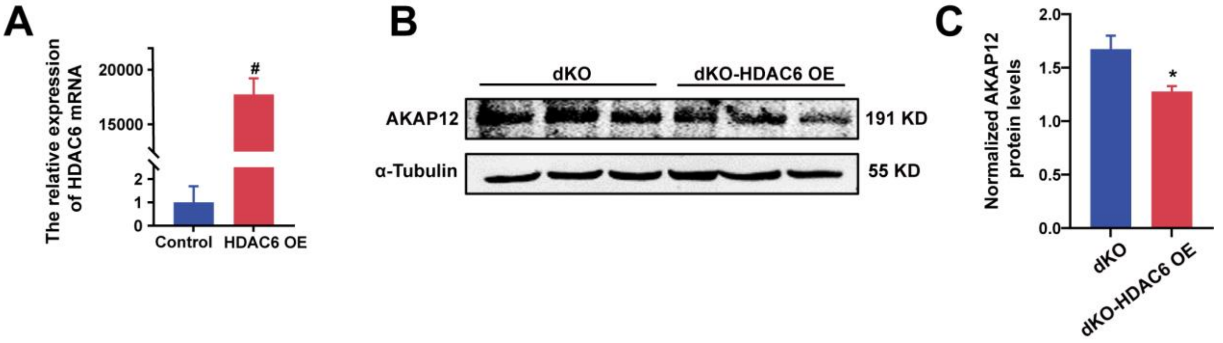

Supplementary Figure S5. HDAC6 mediates G3BPs' inhibition on AKAP12.

A. qPCR analysis of HDAC6 expression levels in control cells and HDAC6-overexpressing cells (OE) 48 h post cell transfection. $n = 3$. **B-C.** Western blot analysis of AKAP12 levels in G3BP dKO cells that were transfected with HDAC6. α-Tubulin was used as a loading control. $n = 3$ from three independent experiments; All data were presented as mean ± SEM; $*p < 0.05$; $\#p < 0.0001$; Student's *t*- test and Bonferroni pairwise comparisons.

Table S4. Seven proteins were highly downregulated in G3BP dKO cells.

Symbol	Gene name	GenBank ID	Abundance Ratio	P value
Atp7b	Copper-transporting ATPase 2	NM_001403709.1	49.8	4.04E-16
Col2a1	Collagen alpha-1(II) chain	NR_177066.1	24.426	4.04E-16
Ak1	Aspartokinase 1	NM_001198790.1	5.171	1.15E-08
Fabp4	Fatty acid-binding protein	NM_024406.4	8.622	6.00E-07
Akap12	A-kinase anchor protein 12	NM_031185.3	3.488	0.0102976
Sdccag8	Serologically defined colon cancer antigen 8 homolog	NR_151682.1	2.409	0.013208
Pdk1	[Pyruvate dehydrogenase (acetyl-transferring)] kinase isozyme 1	NM_172665.5	3.528	0.018369

Table S5. Primers used in this study.

Purpose	Primer name	Primer sequence
sgRNA primers	G3BP1-F	caccGTACTACACTCTGCTGAACC
	G3BP1-R	aaacGGTTCAGCAGAGTGTAGTAC
	G3BP2-F	caccGAAGCTCCCGAGTATTTGCAC
	G3BP2-R	aaacGTGCAAATACTCGGGAGCTTC
	AKAP12-1F	caccGGCCCCACAAGCAGCGGACT
	AKAP12-1R	aaacAGTCCGCTGCTTGTGGGGCC
	AKAP12-2F	caccGTCCACTCCACCATGAACAT
	AKAP12-2R	aaacATGTTCATGGTGGAGTGGAC
	AKAP12-3F	caccGGCGGAAACATTGTTCTCGC
	AKAP12-3R	aaacGCGAGGAACAATGTTTCCGCC
qPCR primers	G3BP2-F	CCGAGGGTTGATGCTAAGCC
	G3BP2-R	CGAATTATTCTCCGGTTGTCAGA
	ATP7B-F	GGGGACGATGCCTGAACAG
	ATP7B-R	TAGCCAACATTGTCGAAGGCG
	COL2A1-F	GGGAATGTCCTCTGCGATGAC
	COL2A1-R	CAGGCGCACCATCTCTGAT
	AK1-F	GCTGCTGTGTGTCTAGTGAAC
	AK1-R	CCTCTCCGATCCAGAGCTGA
	FABP4-F	AAGGTGAAGAGCATCATAACCCT
	FABP4-R	TCACGCCTTTCATAACACATTCC
	AKAP12-F	CTGTCTGCCGTCAATGGTGTA
	AKAP12-R	TGAAGCAGGGATCTGTTCGAT
	SDCCAG8-F	AGGCCCAGGTTGAAAAGGTC
	SDCCAG8-R	CTGAAAGCGCATTTCTCCACA

	PDK1-F	GGACTTCGGGTCAGTGAATGC
	PDK1-R	TCCTGAGAAGATTGTCGGGGA
Dual Fluorescence reporting primers	G3BP2-3'UTR link-F	tcgctagcctcgagtAGTCCCACTGTTGGCAGTTTTG
	G3BP2-3'UTR link-R	gcctgcaggtcgactCTACTAAAGCCAAGATGGTAAAGC
	G3BP2-3'UTR link-Mut1-F	CCCCAATTTGGATTCATATTGTTAATTTATAAAGCTAGACT
	G3BP2-3'UTR link-Mut1-R	TATGAATCCAAATTGGGGAAATAATGTCCACCT
	G3BP2-3'UTR link-Mut2-F	TTTCACATCTGTACAAGCATATATATTTCAAATCC
	G3BP2-3'UTR link-Mut2-R	GCTTGTACAGATGTGAAATATTTACACACATATACACACCTATACAC
	G3BP1-WT1- link-F	aacgagctcgctagcctcgagATGGTTATGGAGAAGCCTAGTCCC
	G3BP1-WT1- link-R	tgcctgcaggtcgactctagaTTCTTCCTCGGATTCCTCTTGA
	G3BP1-WT2- link-F	aacgagctcgctagcctcgagATTCTGAACCTGTTCAGAAGGTCC
	G3BP1-WT2- link-R	tgcctgcaggtcgactctagaTGAAGGACTAAGGGGTCAGGG
	G3BP1-CDS link-Mut1-F	GAgtcctagttcagaCTGAATGACGGGGTGGTGG
	G3BP1-CDS link-Mut1-R	AGtctgaactaggacTCCACGTGGCGGATCTTG
	G3BP1-CDS link-Mut2-F	GATTtacgacCCAAGGCAGTGAATTGCTTGGCT
	G3BP1-CDS link-Mut2-R	TGCCTTGGgtcgtaAATCCCCCTGCCCACGCCA
	G3BP1-CDS link-Mut3-F	ACACAGtcatgtaggCCGGCTCCTGCAGAATGG
	G3BP1-CDS link-Mut3-R	GGcctacatgaCTGTGTGGAGATCAGAGCCAAGC
